# Direct Femtosecond Laser Surface Structuring with Optical Vortex Beams Generated by a q-plate

**DOI:** 10.1038/srep17929

**Published:** 2015-12-10

**Authors:** Jijil JJ Nivas, Shutong He, Andrea Rubano, Antonio Vecchione, Domenico Paparo, Lorenzo Marrucci, Riccardo Bruzzese, Salvatore Amoruso

**Affiliations:** 1 Dipartimento di Fisica, Università di Napoli Federico II, Complesso Universitario di Monte S. Angelo, Via Cintia, I-80126 Napoli, Italy; 2 CNR-SPIN UOS Napoli, Complesso Universitario di Monte S. Angelo, Via Cintia, I-80126 Napoli, Italy; 3 Ultrafast Laser Laboratory, Key Laboratory of Opto-electronic Information Technical Science of Ministry of Education, College of Precision Instruments and Opto-electronics Engineering, Tianjin University, Tianjin 300072, China; 4 CNR-SPIN, UOS Salerno, Via Giovanni Paolo II 132, I-84084 Fisciano, Italy; 5 National Research Council, Institute of Applied Science & Intelligent Systems (ISASI) ‘E. Caianiello’, Via Campi Flegrei 34, 80078 Pozzuoli (NA), Italy

## Abstract

Creation of patterns and structures on surfaces at the micro- and nano-scale is a field of growing interest. Direct femtosecond laser surface structuring with a Gaussian-like beam intensity profile has already distinguished itself as a versatile method to fabricate surface structures on metals and semiconductors. Here we present an approach for direct femtosecond laser surface structuring based on optical vortex beams with different spatial distributions of the state of polarization, which are easily generated by means of a *q-plate*. The different states of an optical vortex beam carrying an orbital angular momentum ℓ = ±1 are used to demonstrate the fabrication of various regular surface patterns on silicon. The spatial features of the regular rippled and grooved surface structures are correlated with the state of polarization of the optical vortex beam. Moreover, scattered surface wave theory approach is used to rationalize the dependence of the surface structures on the local state of the laser beam characteristics (polarization and fluence). The present approach can be further extended to fabricate even more complex and unconventional surface structures by exploiting the possibilities offered by femtosecond optical vector fields.

The crucial role of surface morphology in regulating the properties of a surface accounts for the growing interest in laser surface machining of metals, semiconductors and insulators in view of diverse applications based on the properties of the processed solid surfaces (e.g., optical, mechanical, chemical, biological, wetting, etc.). The use of femtosecond (fs) laser pulses offers advantages in minimizing thermal effects and collateral damage, and the young research field of direct fs laser surface structuring is continuously showing impressive scientific achievements^1^,^2^. In most cases, laser beams with a Gaussian intensity spatial profile and spatially homogeneous distribution of polarization are exploited. A typical aspect of fs laser surface structuring is the spontaneous formation, within the smooth laser beam intensity profile, of periodic sub-wavelength structures preferentially directed along normal to the laser polarization, generally dubbed as ripples or laser-induced periodic surface structures (LIPSS), without any use of beam shaping or holography^1^,^2^,^3^. More recently, attention is also focused on other supervening quasi-periodic surface microstructures with orientation parallel to laser polarization, named as grooves, which form on semiconductors (Si and InP, e.g.) irradiated by a large number of laser pulses and at higher fluence than ripples^4^,^5^,^6^,^7^.

Complex polarization patterns in a single laser beam can offer the possibility of generating complex surface structures. Polarization is an intrinsic and key element of light and the use of laser beams with a spatially inhomogeneous state of polarization (SoP) is bringing out novel breakthroughs on the horizon of fs laser material processing^8^,^9^. The possibility to use vector optical fields to directly fabricate surface microstructures is emerging as a fascinating possibility and is proposed as an effective method for vector beams characterization^10^,^11^,^12^ as well as for unconventional surface structuring^13^,^14^,^15^,^16^. One interesting possibility to obtain an unconventional distribution of SoP is provided by a beam with non-vanishing orbital angular momentum^17^. In this paper, we report on direct fs laser surface structuring using an optical vortex (OV) beam, generated by a *q-plate* and carrying an orbital angular momentum ℓ = ±1 (per photon, in units of ħ), with different spatial distributions of its SoP. The different states of the OV beam are used to demonstrate the fabrication of various regular surface patterns. The spatial features of the rippled and grooved surface structures are correlated with the optical vortex beam SoP. Moreover, numerical simulations based on a scattered surface wave theory are exploited to rationalize the relationship between the local state of the laser beam polarization and fluence and the surface structures.

## Results

### Principle

**Figure 1:**
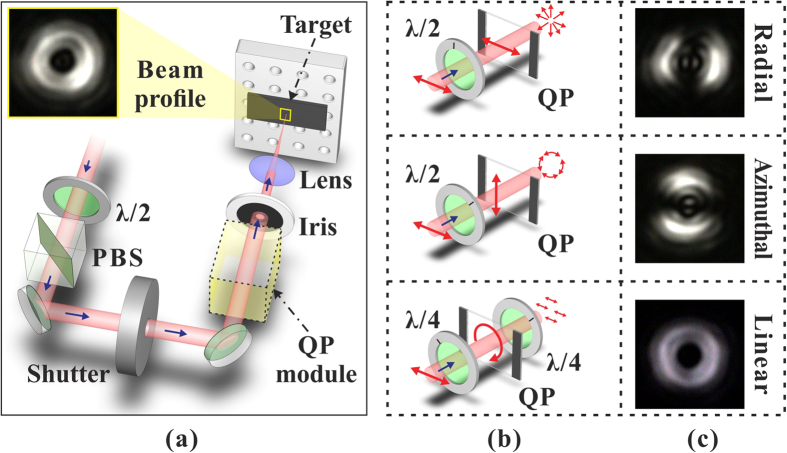
(**a**) Direct fs laser surface structuring with OV beams generated by a *q-plate*. (**b**) Three examples of the q-plate module configuration used to generate an OV beam with radial, azimuthal and linear SoP, respectively. λ/2 and λ/4 are half and quarter wave plates, respectively. (**c**) Optical patterns registered after a horizontally-oriented polarizing filter showing the radial, azimuthal and linear SoP of the OV beam.

The concept of our method is shown in Fig. 1, and the main element of our approach is the *q-plate* module. The q-plate (QP) is an optical device based on liquid crystal technology that is capable of generating and manipulating light beams carrying orbital angular momentum (OAM)^18^,^19^. The QP essentially acts as a birefringent wave plate with an inhomogeneous distribution pattern of the local optical axis in the transverse plane. This pattern of the optical axis distribution is defined by a semi-integer topological charge *q*. Here we exploit a QP with *q* = +1/2 that allows generating an OV beam carrying an OAM ℓ = ±1. The input beam is provided by a linearly polarized Ti:Sa laser source producing ≈35 fs laser pulses with a Gaussian spatial intensity distribution. After the QP module, the spatial intensity distribution of the beam presents a central region of zero intensity (due to undefined phase on the beam axis), a principal intense ring and several secondary rings at increasing radial distance from the axis^19^. The central part of this beam is spatially filtered with an iris and focused on the silicon target surface by a lens. The final beam irradiating the target surface is characterised by an annular spatial profile, as shown in the upper right inset of Fig. 1(a). The SoP of the OV beam can be easily manipulated by means of wave plates whose optical axis is appropriately aligned with respect to the QP optical axis. Figure 1(b) illustrates three examples of the QP module configuration used to generate radial, azimuthal and linear SoP. The SoP is checked by using a horizontally-oriented polarizing filter and a beam profiler, as shown in Fig. 1(c). For the experiments, the OV pulse energy, *E*_*0*_, is varied by means of a system of half wave plate and polarizing beam splitter, while the number of pulses hitting the target surface, *N*, is selected by an electromechanical shutter. The system allows investigating the surface structuring with OV annular beams characterised by different SoP. The experimental procedure is detailed in the section Methods.

### Surface patterns produced by OV beam with different SoP

**Figure 2:**
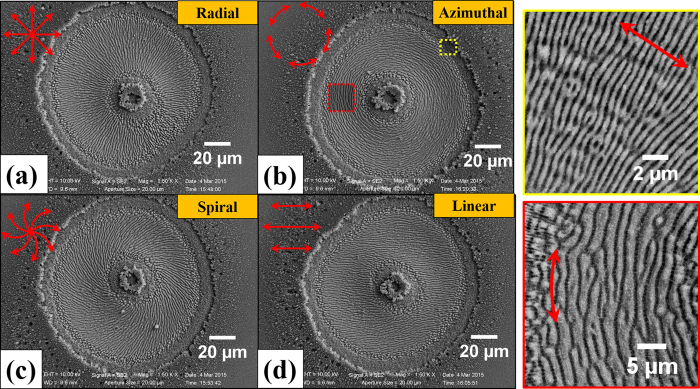
Examples of the surface structures developed on the silicon target after an irradiation sequence of *N* = 100 pulses at an energy *E*_*0*_ = 48 μJ with different SoP: (**a**) radial, (**b**) azimuthal, (**c**) spiral and (**d**) linear. The two panels on the right illustrate the fine morphology of the surface structure for the azimuthal SoP: peripheral regions (yellow box) at the outer edges of the annular OV beam are namely characterised by subwavelength ripples oriented along the normal to the local laser polarization (double-ended arrow), while the internal region of the OV beam (red box) presents microgrooves preferentially directed along the local laser polarization. The fine surface texture observed for the other SoP shows the same characteristic features.

**Figure 3:**
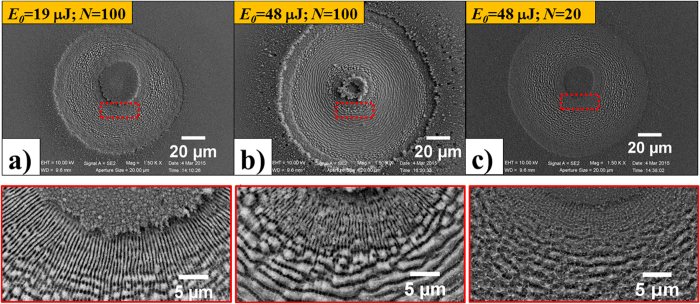
SEM images of the surface structures on the silicon target after an irradiation sequence of *N* pulses with azimuthal SoP for two different values of the pulse energy and number of pulses: (**a**) *E*_*0*_ = 19 μJ, *N* = 100; (**b**) *E*_*0*_ = 48 μJ, *N* = 100; (**c**) *E*_*0*_ = 48 μJ, *N* = 20. The lower panels are zoomed views of the areas indicated by the red dashed box in the corresponding upper SEM images illustrating the dependence of the fine morphology of the surface structure on the laser pulse energy *E*_*0*_ and number of irradiating pulses *N*.

We studied fs direct laser structuring of silicon because it is a case study for surface structures formation and the most relevant semiconductor material. Moreover, its physical properties are well-known and available for modeling. Figure 2 reports examples of SEM images of the target surface illustrating the complex surface patterns that can be generated by fs OV laser pulses with different SoP. The irradiating conditions are *N* = 100 and *E*_*0*_ = 48 μJ, which corresponds to a peak fluence *Φ*_*p*_ = 0.53 J/cm^2^. The resulting surface presents various annular regions characterised by different surface morphologies. In all cases, a central structure forms in the inner region of the OV beam with almost null intensity, which is constituted by an assembly of a large number of nanoparticles. A layer of nanoparticles is also present in the outer area of the OV beam. This indicates that random nanoparticles assembled nanostructures form in the central and external areas of the OV beam where the local fluence is lower than the ablation threshold. The ablated annular crater presents an inner ring-shaped region characterised by micron sized grooves preferentially aligned along the local beam polarization. A zoomed view of these grooves is presented in the inset (red box) of Fig. 2 showing part of this region for azimuthal SoP. The grooved region extends over the most part of the ablation crater in all the cases shown in Fig. 2, and is surrounded by two adjacent, narrow ring-shaped areas (in either sides) with a characteristic texture of subwavelength ripples aligned perpendicular to the laser polarization. The other inset (yellow box) of Fig. 2 shows a zoomed view of the ripples for the azimuthal SoP. Similar features are observed for other SoP of the OV beam. The analysis of the surface patterns of Figs 2 and 3 clearly demonstrates that the QP module constituted by a q-plate with *q* = +1/2 and a half wave plate allows fabricating radial, azimuthal and all sorts of intermediate spiral textures as that shown in Fig. 2(c), by appropriate tuning of the half wave plate axis alignment with respect to the q-plate axis. Moreover, linear surface patterns can be realized within a ring-shaped region by exploiting two quarter wave plates (see lower panels of Figs 1(b,c) and 2(d)).

Both the overall width of the ablated crater and the thickness of the rippled and grooved annular regions depend on the spatial distribution of the OV laser pulse fluence, *E*_*0*_, as well as on the number of pulses, *N*. As an example, Fig. 3 reports SEM images of the target surface after an irradiation sequence of *N* pulses with azimuthal SoP for two different values of the pulse energy and number of pulses, namely (a) *N* = 100, *E*_*0*_ = 19 μJ; (b) *N* = 100, *E*_*0*_ = 48 μJ and (c) *N* = 20, *E*_*0*_ = 48 μJ. The lower panels show zoomed views of the surface corresponding to the regions identified by the red dashed boxes. At the lower energy *E*_*0*_ = 19 μJ for *N* = 100 (Fig. 3(a)), ripples dominate the surface texture of the annular crater, while grooves rudiments decorate the underlying ripples. At the same number of pulses *N* = 100 and higher energy *E*_*0*_ = 48 μJ (Fig. 3(b)), instead, well-developed grooves cover the inner part of the annular crater and clear, sharp spatial transitions between the external rippled areas and the central grooved region occur. This, in turn, suggests the existence of a well-defined fluence threshold for the transition from ripples to grooves, *Φ*_*th,G*_. Moreover, it also supports the idea that appropriate shaping of SoP and fluence profile can be used to fabricate diverse complex surface patterns. The comparison between Fig. 3(c,b) corresponding to *N* = 20 and *N* = 100, at the same energy *E*_*0*_ = 48 μJ, allows addressing the role of the number of pulses. One can observe that at the lower value of *N*, at high energy, the surface texture already shows characteristic features of grooves which are reinforced and well-developed as the pulse number increases.

### Features of the surface patterns produced by OV beam and formation mechanisms

Here, we illustrate the main characteristics of ripples and grooves produced on silicon with OV beams. In particular, variation of the fluence threshold for the formation of grooves, model predictions for the structures generation and dependence of the period of the formed surface structures on *E*_*0*_ and *N* will be addressed. The experimental data refer to an azimuthal SoP, for which a thorough analysis was carried out, but similar behavior was also observed for other SoP. It is worth mentioning here that ripples produced by Gaussian beams are extensively studied, and various mechanisms are considered to rationalize their formation, as for example excitation of surface scattered waves and surface plasmon polaritons, self-organization of surface instabilities, etc. (see e.g. Refs. 1, 2, 3and references quoted herein). Nevertheless, no general consensus has been reached yet, while grooves formation mechanisms have not been thoroughly analysed so far. We believe that a detailed analysis of the surface texture in the passage from the rippled to the grooved region of the irradiated target reported hereafter can provide interesting insights in the grooves formation mechanisms.

### Fluence threshold for grooves formation

**Figure 4:**
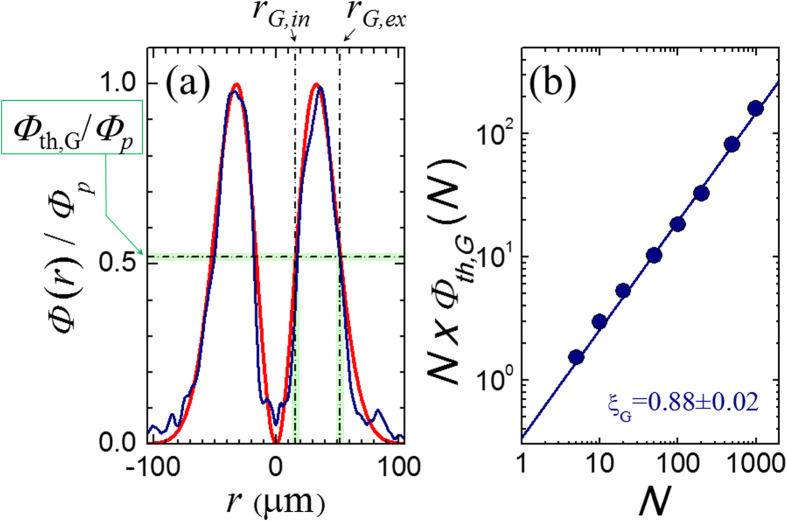
(**a**) Example of the spatial profile of the annular beam as a function of the coordinate *r* along an axis coincident with a diameter of the OV beam (*r* = 0 marks the center of the OV beam). The blue line is the experimental profile, while the red line is a fit. (**b**) Variation of the threshold fluence *Φ*_*th,G*_ as a function of the number of pulses *N* displayed on a log-log plot of *N* × *Φ*_*th,G*_(*N*) vs *N.* The uncertainty on the experimental data points is within the size of the symbols. The line is a fit to a power law dependence typical of an incubation behavior with a slope ξ_G_ = (0.88 ± 0.02). The data refer to an azimuthal SoP.

The fluence threshold *Φ*_*th,G*_ for grooves formation varies with the number of pulses *N*. As the surface texture depends on the local OV beam fluence, the variation of *Φ*_*th,G*_ with *N* is obtained by measuring the values of the radii *r*_*G,in*_ and *r*_*G,ex*_ of the two circles delimiting the grooved area formed after irradiation with different values of the pulse number *N*, and estimating the corresponding value of the fluence threshold *Φ*_*th,G*_ from the spatial profile of the OV pulse fluence *Φ*(*r*), as shown in Fig. 4(a). In Fig. 4(a), the blue line shows the experimental fluence profile, normalized to the peak fluence *Φ*_*p*_, while the red line is a fitting curve^17^, as described in the section Methods. The estimated values of *Φ*_*th,G*_ are reported in Fig. 4(b), which shows that the experimental data are well described by a linear dependence on a log-log plot of (*N* × *Φ*_*th,G*_) vs *N*, thus supporting a power law dependence of the threshold fluence typical of an incubation behavior^20^:


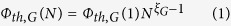


where *Φ*_*th,G*_(1) = (0.34 ± 0.04) J/cm^2^ is the threshold fluence for *N* = 1 and *ξ*_*G*_ = (0.88 ± 0.02) is the incubation factor. The incubation effect is known to reduce the multiple-pulse ablation threshold as the number of pulses increases as a consequence of defect creation and related feedback effects for laser-induced surface modification^3^,^20^. By studying the dependence on *N* of the threshold fluence for surface modification of silicon induced by fs pulse with a Gaussian intensity profile, Bonse *et al.* reported an incubation factor of ≈0.84^20^, which is consistent with our estimate. Incubation behavior has been reported earlier for laser induced damage and ripples formation of different materials^3^,^20^,^21^, but it was never associated to the generation of grooves. The existence of an incubation effect for grooves formation suggests that the dynamic evolution of the target surface during multi-pulse exposure to laser pulses is also a key factor for the creation of these surface structures.

### Mechanisms of ripples and grooves formation

Recently, we showed that the Sipe-Drude approach is able to explain fine morphological features of ripples and grooves, namely bending and bifurcation phenomena, produced during irradiation of silicon with a fs laser beam with a Gaussian intensity profile^4^. The Sipe-Drude model is an extension of the surface-scattered wave theory of Sipe *et al.*^22^,^23^ which takes into account the effects of the carrier-dependent variation of the dielectric permittivity ε of the target surface induced by fs laser pulse irradiation^4^,^24^,^25^,^26^. As described in the section Methods, this approach interprets the generation of periodic surface structures in terms of a spatially-modulated pattern of energy deposition on a rough target surface. In particular, we evaluate the spatially-modulated pattern of the energy deposition in the real space by considering the 2D-IFT map 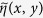 corresponding to local features of the OV laser beam, i.e. the value of the fluence and direction of polarization in the area of the observation point on the irradiated target.

**Figure 5:**
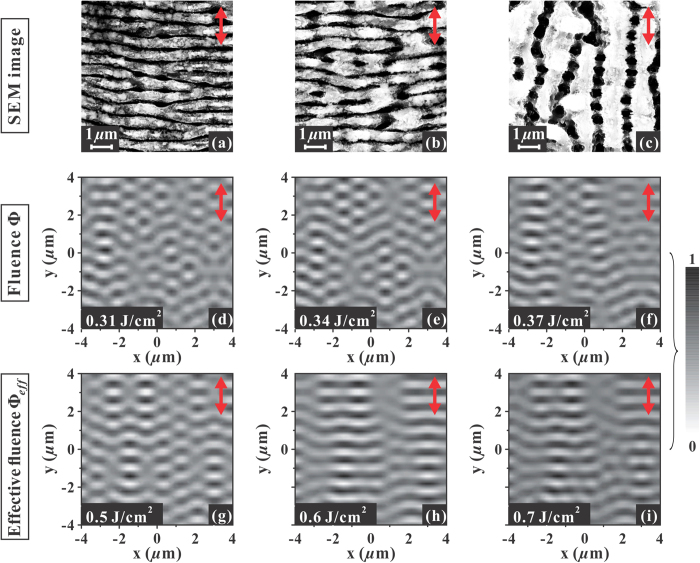
Upper panels: SEM images of the surface structures on the silicon target after an irradiation sequence of *N* = 100 pulses with azimuthal SoP in the transitional region from ripples to grooves: panels (**a–c**) show ripples and grooves aligned perpendicular and parallel to the local laser polarization, respectively. Panel (**b**) corresponds to the transition region where grooves rudiments form on top of the underlying ripples and corresponds to a local value of the laser fluence *ϕ* ≈ *Φ*_*th,G*_(1) = 0.34 J/cm^2^. Panels (**d,f**) report representative 2D-IFT maps of the efficacy factor calculated for three different values of the local laser fluence *ϕ*: panel (**e**) corresponds to *ϕ*  = 0.34 J/cm^2^, while the other two side maps in panels (**d,f**) are relative to values of the fluence differing by ≈ ± 10% with respect to panel (**e**). Panels (**g,i**) show representative 2D-IFT maps of the efficacy factor calculated for three different values of the local laser fluence value *ϕ*_*eff*_ (see Eq. (2)): panel (**h**) corresponds to *ϕ*_*eff*_ = 0.6 J/cm^2^, while the other two size maps in panels (**g**) and (**i**) are relative to values of the fluence below (*ϕ*_*eff*_  = 0.5 J/cm^2^) and above (*ϕ*_*eff*_  = 0.7 J/cm^2^) the one of panel (**h**), respectively. In all the panels, the double-headed arrow indicates the local direction of the laser polarization. The 2D-IFT maps are normalized to their own corresponding maximum intensity according to the scale shown on the right.

Figure 5(a–c) reports SEM images of the structures formed on the silicon target surface after an irradiation sequence of *N* = 100 pulses with azimuthal SoP. The local direction of the laser polarization is indicated by a double-headed arrow. These images were registered close to the transitional region from ripples to grooves, and illustrate the variation of the surface morphology while passing from a region at a fluence lower than *Φ*_*th,G*_(1) (Fig. 5(a)), where sub-wavelength ripples aligned normal to local laser polarization are observed, to a region at a fluence larger than *Φ*_*th,G*_(1) (Fig. 5(c)), characterised by well-developed grooves preferentially directed parallel to local laser polarization. Figure 5(b) corresponds to a local value of the fluence *ϕ* ≈ *Φ*_*th,G*_(1) and shows grooves rudiments forming on the top of ripples. Interestingly, in Fig. 5(c) the underlying remnants of ripples can be observed in the gaps between adjacent grooves. The width of the deep wrinkles separating the remnants of ripples increases from Fig. 5(a–c), indicating that a progressive reduction of the thickness of the underlying ripples is also associated to grooves formation.

We turn now to model predictions. Figure 5(d–f) report representative 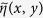 gray-scale maps of the efficacy factor illustrating the spatial modulation of the energy-deposition calculated for three different values of the local laser fluence *ϕ*. The maps are evaluated in different positions of the OV beam radial profile in order to take into account the spatial variation of the beam fluence and the characteristics of the corresponding surface structure. For each map, the intensity is normalized to its own maximum value according to the scale shown on the right. In the 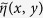 maps of Fig. 5, the local direction of the laser polarization is always aligned along the *y*-coordinate as indicated by the double-headed arrow. Figure 5(e) shows the 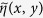 map corresponding to a local value of the laser fluence *ϕ* = 0.34 J/cm^2^, which corresponds to the threshold for grooves formation extrapolated to a single-shot irradiation condition, *Φ*_*th,G*_(1). The other two maps in panels (d) and (f) are relative to values of the fluence *ϕ* differing by ≈ ± 10% with respect to panel (e). All the three maps show a quasiperiodic distribution of the laser deposited energy preferentially directed along the normal to the laser polarization. This should lead to digging out deeper wrinkles in the regions of higher absorption leading to formation of ripples. Nevertheless, the variation of local fluence *ϕ* going from panel (d) to (e) leads only to minimal changes to the predicted pattern, without showing a clear transition suggestive of the possible formation of grooves. Instead, the patterns remain rather irregular resembling the rudiments of ripples typically observed at low number of pulses^4^,^14^.

Previous experimental results indicate that a multi-pulse feedback mechanism occurs in fs surface structuring leading to ripples formation^5^,^6^,^14^. Consequently, this suggests a possible direct influence of the mechanisms leading to the incubation phenomenon discussed above on the features of the generated surface pattern. Sipe-Drude model cannot directly simulate the evolution of the surface under multi-pulse irradiation conditions typically used in experiments. However, the incubation effect progressively reduces the threshold fluence for grooves formation as the number of pulses *N* increases (see Fig. 4(b)), thus suggesting that a varying level of effective excitation is associated with the cumulative effect of pulse number *N*. We propose to introduce such an effect within the Sipe-Drude model by considering an effective fluence *ϕ*_*eff*_ rescaled with respect to the experimental single-shot threshold fluence for grooves formation *Φ*_*th,G*_(1) in the same proportion as the actual local fluence *ϕ* scales with the threshold fluence for *N* pulses *Φ*_*th,G*_(N). This reads *ϕ*_*eff*_ /*Φ*_*th,G*_ (1) = *ϕ* /*Φ*_*th,G*_(N), and consequently:


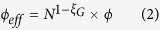


Therefore, for *ϕ* = 0.34 J/cm^2^ and *N* = 100, the value of the effective fluence for grooves formation is *ϕ*_*eff*_ ≈ 0.59 J/cm^2^. We can use Eq. (2) to associate an effective value of the local fluence to any position along the beam radius. Figure 5(h) reports the 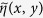 map of the efficacy factor illustrating the spatial modulation of the energy-deposition calculated for a local fluence value *ϕ*_*eff*_ = 0.6 J/cm^2^. The other two maps in Fig. 5(g,i), respectively, correspond to local fluence values below (*ϕ*_*eff*_ = 0.5 J/cm^2^) and above (*ϕ*_*eff*_  = 0.7 J/cm^2^) the one of Fig. 5(h) relative to the grooves formation threshold. Therefore, the three maps in panels (g)-(i) of Fig. 5 visualize the local energy modulation patterns corresponding to three different locations in the OV beam spot and specify the conditions for a local value of the below (g), at (h) and above (i) the threshold for grooves formation, respectively. The map of Fig. 5(g) (*ϕ*_*eff*_  = 0.5 J/cm^2^) shows an energy modulation pattern of relatively linear, quasi-periodic thin strips perpendicular to the laser polarization with a subwavelength period. This agrees well with the pattern of ripples observed in Fig. 5(a) and characteristic of the region of the sample irradiated at a fluence below the grooves formation threshold. The progressive increase of *ϕ*_*eff*_ to 0.6 J/cm^2^ (Fig. 5(h)) and 0.7 J/cm^2^ (Fig. 5(i)) leads to 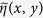 maps characterised by the presence of larger, micron-sized secondary structures in form of lower intensity ribbons that overlap the quasi-periodic ripple pattern. The ribbons are preferentially aligned along the laser polarization and are characterised by an amplitude ≈3 times lower than the maximum value (in black). Moreover, the variation of the intensity along the ribbons is almost negligible. These feature becomes more important as the value of *ϕ*_*eff*_ increases, and we deem that it is associated with the progressive formation of grooves aligned along the laser polarization observed in the region of the annular, irradiated target surface with larger values of the local fluence. Thus, our findings show that two orthogonal, quasi-periodic patterns coexist in the map of the efficacy factor 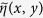 at larger excitation level: a secondary modulation pattern of the absorbed energy is superimposed over the characteristic, primary pattern with a period close to the incident laser wavelength associated to the ripples. The secondary modulation forms linear structures preferentially directed along the laser polarization with a period of few microns. Moreover, the secondary pattern tends to wipe out the primary modulation, which only survives in the regions of higher amplitude of the efficacy factor. The close correspondence between the morphology of the absorbed laser energy predicted by the Sipe-Drude model and the characteristics of the surface pattern observed in Fig. 5(c) is suggestive of a tight connection between the mechanisms of grooves formation and the modulation of the efficacy factor 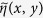 represented in Fig. 5(h,i). The quasi-periodic pattern of the secondary ribbons indicates a spatial redistribution of the absorbed energy leading to the development of regions where the absorbed energy is not high enough to induce an effective ablation, while being able to melt the surface nanostructures present in these regions^4^. The melting of such surface structures can progressively lead to the formation of the grooves covering the underlying ripples. In addition, the modulation of the energy in the gap between the ribbons observed in Fig. 5(h,i) points to the formation of small areas of maximum energy absorption located close to ripples, which can explain the gradual increase of the separation distance between the underlying ripples remnants observed in Fig. 5(a) as consequent to a spatially selective ablation in these areas.

### Spatial period of ripples and grooves

**Figure 6:**
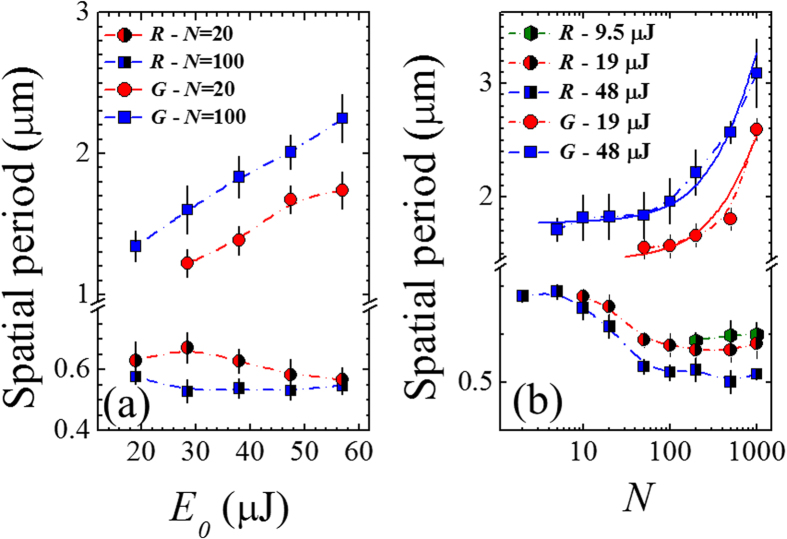
(**a**) Variation of the spatial period of ripples and grooves as a function of the laser pulse energy *E*_*0*_, for two different values of the laser pulse number: *N* = 20 and *N* = 100. (**b**) Variation of the spatial period of ripples and grooves as a function of the number of pulses *N* for three different values of the laser pulse energy: *E*_*0*_ = 9.5 μJ, *E*_*0*_ = 19 μJ and *E*_*0*_ = 48 μJ. The two solid lines represent linear fits to the experimental data (note that data are reported on a lin-log plot). The legend *R* and *G* indicate ripples and grooves, respectively.

Further analysis was also carried out on the produced surface structures to investigate the variation of the ripples and grooves spatial period with laser pulse energy *E*_*0*_ and number of pulses *N*. Figure 6 reports the ripples and grooves spatial period as a function of *E*_*0*_ (panel (a)) and *N* (panel (b)). As the exact value of the period slightly varies with the specific location, an average value of the period was estimated and the observed variability was indicated as an error bar. Considering first the ripples, Fig. 6(a) shows a weak dependence of the ripple period on the laser pulse energy *E*_*0*_, at a fixed number of laser pulses (namely, *N* = 20 and 100). In particular, it seems to reduce very slightly as *E*_*0*_ increases. Instead, Fig. 6(b) indicates an important effect of the number of pulses on the spatial period, which progressively reduces for 10 < *N* < 100, and then reaches a plateau at larger values of *N*, at *E*_*0*_ = 19 μJ and 48 μJ. The decrease of the ripples spatial period with the number of pulses has been reported earlier^5^,^26^,^27^,^28^, while the dependence on pulse energy is still scarcely investigated^27^. Interestingly, at lower laser fluence, e.g. *E*_*0*_ = 9.5 μJ, ripples with a period of ≈0.6 μm are only observed for *N* ≥ 200, while no well-developed grooves are present in such experimental conditions even at *N* as large as 1000.

The spatial period of grooves shows a completely different dependence on *E*_*0*_ and *N*. In particular, Fig. 6(a) shows that the period increases linearly with the pulse energy *E*_*0*_ in the investigated range, for both *N* = 20 and *N* = 100. Figure 6(b) indicates that, at fixed pulse energy, the grooves period progressively rises with *N*. Moreover, well-developed grooves only form at a larger number of pulses for lower energy. Our experimental findings on the grooves period dependence on *N* agrees well with recent observations of Tsibidis *et al.*^7^, who reported an approximately linear increase of the grooves periodicity with *N* during irradiation of silicon with 470 fs, 800 nm laser pulses with Gaussian intensity spatial profile at a laser fluence of 0.7 J/cm^2^. In such a report the analysis was limited to a number of pulses 20 < *N* < 100, here we observe that the dependence of the spatial grooves period remains approximately linear even for *N* as high as 1000, as shown by the two solid lines in Fig. 6(b).

## Discussion

The use of OV beams opens numerous novel possibilities in the field of direct fs laser surface structuring. Indeed, by exploiting an OV beam generator based on a q-plate we demonstrated the possibility to tune the SoP of the OV beam and extend the form of regular surface patterns that can be fabricated by direct fs laser irradiation. Two main surface structures have been observed depending on the level of excitation of the silicon target surface. At low level of excitation, subwavelength ripples preferentially oriented in a direction normal to the local laser polarization are generated, while at larger excitation level a regular pattern of micron-sized grooves aligned along the local laser polarization is formed.

**Figure 7:**
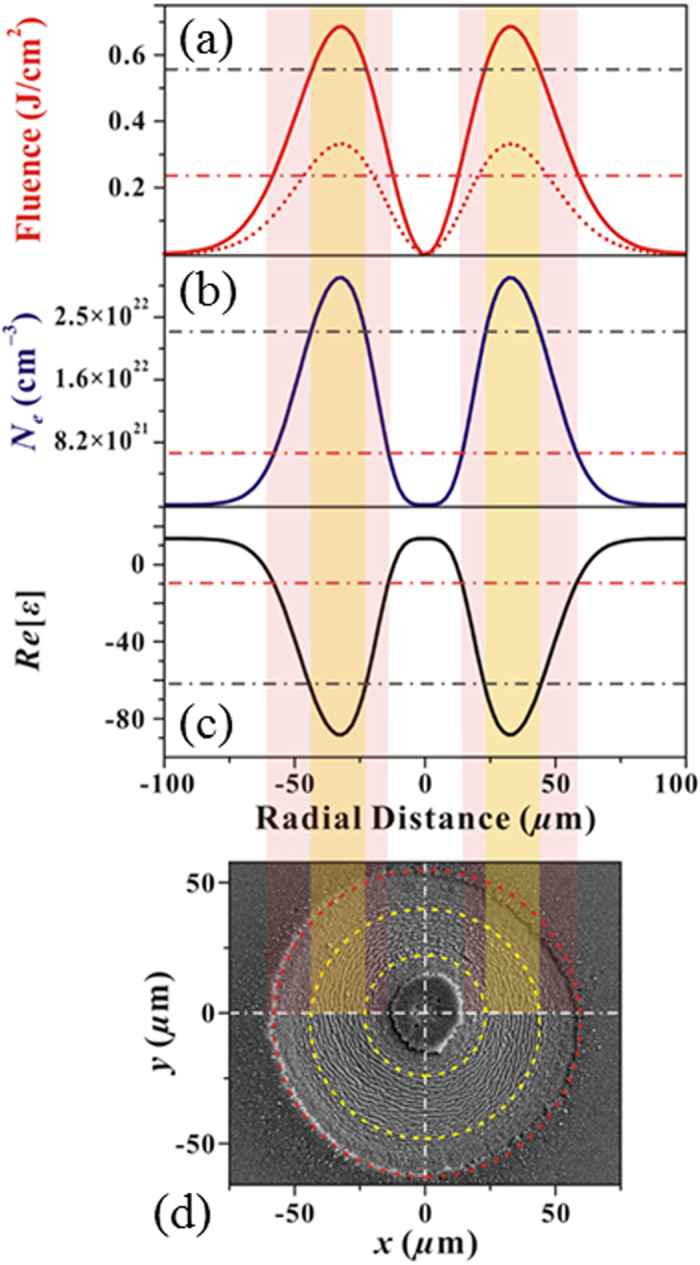
Dependence of the surface structures on the OV laser beam spatial intensity profile. Panel (**a**) reports the OV fluence profile for *E*_*0*_ = 29 μJ and w_0_ = 46 μm (dashed curve) and the corresponding distribution of the effective fluence for *N* = 100 (solid curve). Panels (**b,c**) show the spatial profiles of the electron density *N*_*e*_ and real part of the dielectric permittivity *Re*[*ε*] predicted by two-temperature model and free-carrier number density equations, respectively. Panel (**d**) reports a SEM image of the target surface after an irradiation sequence of *N* = 100 laser pulses at *E*_*0*_ = 29 μJ. The circles and color stripes marks the distinct regions with ripples and grooves.

Figure 2 shows examples of the various surface structures that can be fabricated, while Fig. 7 exemplifies the close relationship between the surface structure features and the spatial profile of the OV beam. The distinct spatial separation between ripples and grooves within the annular crater formed on the target surface and the surface structure features illustrated above points to a strict relationship with the level of excitation achieved in the different regions of the target surface after OV beam irradiation with a series of *N* pulses at energy *E*_*0*_. Even if no general consensus has been reached yet on the mechanisms of ripples formation, the most widely accepted scenario explains their generation in terms of the interference between the incident laser beam and a surface scattered electromagnetic wave induced by surface roughness. The resulting modulation of the absorbed energy is typically characterised by a subwavelength period, which eventually results in selective ablation and formation of ripples oriented normal to the laser polarization. Here we consider this interpretation and simulate the local spatial patterns of absorbed energy by means of the Sipe-Drude model.

We recall here that the predictions of the Sipe-Drude model are coherent with our experimental findings when the incubation effect occurring during multi-pulse laser irradiation is taken into account (see Fig. 5). In particular, we have inserted this aspect in the model by appropriate scaling of the effective, local fluence with the number of pulses *N* (see Eq. 2). In the following, we discuss first at which extent our approach is able to explain the characteristic dependencies of ripples features on the experimental parameters *E*_*0*_ and *N*. Then, we discuss the possible insights it can provide on mechanisms involved in the generation of the microgrooves oriented along the laser polarization that develops at higher excitation level.

Fig. 7(a) reports the OV beam spatial profile for *E*_*0*_ = 29 μJ, which corresponds to peak fluence of 3.2 J/cm^2^ (dashed curve). As an example, the SEM image obtained in this experimental conditions, for *N* = 100 pulses, is shown in Fig. 7(d). The spatial profile of the effective fluence for *N* = 100 estimated by using Eq. (2) is also reported as solid curve in Fig. 7(a). Figure 7(b) shows the spatial profile of the electron density *N*_*e*_ obtained by solving two-temperature model and free-carrier number density equations^4^,^24^,^25^ for the effective fluence profile of Fig. 7(a), while Fig. 7(c) reports the corresponding spatial variation of the real part of the complex relative dielectric permittivity *ε*. The comparison with the SEM image of Fig. 7(d) allows identifying the distinct levels of excitation associated to the different regions of the silicon surface irradiated by the OV beam. As observed in Figs 2,3,and 7(d), moving outwards from *x* = 0 one encounters internal and external rippled annular regions of lower fluencies separated by an intermediate region of higher fluencies occupied by grooves.

Considering first ripples, the interference model of Sipe-Drude predicts that, at normal incidence, the ripples period scales with *ε* as^1^,^3^:


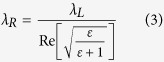


where λ_*L*_ is the incident laser wavelength and *Re*[***z***] indicates the real part of the complex number ***z***. As *ε* depends on the local fluence (see e.g. Fig. 7), a variation of the ripple period with the radial position is expected, at a fixed pulse energy *E*_*0*_. In particular, taking into account the spatial profiles of *ε* and Eq. (3), the theory predicts that a larger period λ_*R*_ is associated to regions of higher fluence. This is indeed experimentally observed, λ_*R*_ varying from ≈0.55 μm for the ripples in the lower energy part of the rippled annular region to ≈0.75 μm for the ripples located closer to the transitional edge towards grooves induced by higher values of the local fluence. We also notice that, for any fixed number of pulses *N*, the ripples only form in the regions where the fluence values are lower than the threshold fluence for the grooves formation *Φ*_*th,G*_(*N*). Since *Φ*_*th,G*_(*N*) progressively reduces with *N* (i.e. 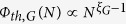), the rippled region of the irradiated area tends to shrink in agreement with our experimental observations (see for example the SEM images of Fig. 3(b,c)). As a consequence, as *N* rises the ripples will form in regions of the OV beam spot characterised by a gradually lower value of the effective fluence. This, in turn, leads to a progressive reduction of the ripples period with *N* that is coherent with our experimental observations (see Fig. 6(b)). The gradual decrease of the ripple period with *N* has been reported earlier for silicon irradiated by fs laser beams with a Gaussian intensity spatial profile for low number of laser pulses (2 ≤ *N* ≤ 8) by Tsibidis *et al.*^26^ and for *N* ≤ 100 by Han *et al.*^27^. Interestingly, we observe that the reduction with *N* is then followed by a plateau at large number of pulses. This is explained by a progressive saturation of the electron density and of the corresponding value of the dielectric permittivity at the high excitation level associated to a large number of laser pulses. Moreover, at very low energy (see e.g. *E*_*0*_ = 9.5 μJ in Fig. 6(b)) ripples are prevalently formed in the irradiated area for *N* ≥ 200 with a period almost independent of the pulse number, in the investigated range of *N*.

Finally, at a fixed number of pulses *N*, an increase of the laser pulse energy *E*_*0*_ results in a rise of the peak fluence and the conditions for ripples formation is only fulfilled in the low fluence regions of the OV beam (*Φ*  ≤ *Φ*_*th,G*_(*N*)). Hence, the two internal and external rippled annuli are progressively located closer to and farther from the central singularity, respectively, while their thickness gradually narrows, in agreement with our experimental findings (see e.g. Fig. 3(a,b)). Moreover, the external rippled ring is always larger than the internal one, due to the different slopes of the spatial intensity profile in the two regions of the OV beam (see e.g. Fig. 7(a)). This also implies that the range of values of the fluence for ripples formation remains almost unchanged as the pulse energy varies, thus explaining the weak dependence of the average ripple period on *E*_*0*_ observed in Fig. 6(a).

As for grooves, the interference model of Sipe-Drude predicts the generation of a secondary modulation of the absorbed energy with a characteristic micron-scale spatial period parallel to the local laser polarization, at high excitation level. Strikingly, a good agreement with experimental observations is achieved by using a simple scaling law of the effective fluence empirically taking into account the incubation effect observed for the variation of the fluence threshold for grooves formation (see Fig. 5). We observe a rather different dependence on both OV beam energy and number of pulses. In particular, the spatial period of grooves shows an approximately linear increase with *E*_*0*_ and *N* (see Fig. 6). Such a dependence cannot be explained by the electromagnetic interference effect of the Sipe-Drude model, since a saturation of both electron density and dielectric permittivity occurs at higher excitation level. Therefore, we deem that the secondary modulation of the absorbed energy acts as a *seed* that triggers the formation of micro-grooves along the direction of laser polarization, but more complex effects related to the dynamics of the melted surface material located in these regions characterised by lower absorption are involved. Such an aspect has been very recently addressed by Tsibidis *et al*.^7^ by means of a hybrid model complementing the electromagnetic interference effects with melt hydrodynamics, which predicts an approximately linear dependence of the grooves spatial period with the number of pulses for 20 ≤ *N* ≤ 100, for a fs laser pulse with a Gaussian intensity profile. Such a study only addressed the role of *N*, while the effect of the pulse energy on the grooves period is still scarcely investigated. Nevertheless, our experimental results indicate that the grooves period scales almost linearly with both *E*_*0*_ and *N* (even for *N* as high as 1000), thus suggesting that the overall excitation level affects the grooves formation and evolution dynamics.

In conclusion, we have experimentally investigated direct surface structuring of silicon with fs OV beams. The good correspondence between predictions of the Sipe-Drude model with the insertion of incubation and the experimentally observed surface structures suggests that this approach is able to retain the main physical features influencing ripples and grooves formation in silicon irradiated by fs laser pulses. In particular, the surface develops a texture according to the excitation level achieved at that location, which depends on the local laser pulses fluence and number of laser pulses, and on the local SoP. Subwavelength ripples aligned along a direction normal to the laser polarization forms in the region of low fluence, while micron sized grooves are generated in the region of higher fluence, which are preferentially aligned along the direction of the laser polarization. Moreover, remnants of subwavelength ripples are present in the gaps between the grooves. Our findings single out the possibility to generate complex surface structures with regular pattern of subwavelength ripples, microgrooves or mixed systems of structures by appropriate tuning of the level of excitation achieved through an appropriate selection of the fluence and number of laser pulses. While our investigation was limited to SoP generated with a q-plate with a topological charge *q* = +1/2, preliminary results with higher values of *q* indicate that OV beams with even more complex SoP can be designed and used to fabricate still more complex surface micro-structures. Moreover, since the formation of periodic surface structures seems to be ubiquitous to fs laser irradiation of solid targets, the method can be directly extended to other materials of interest as preliminary findings indicate. Both these aspects will be subject of future communications.

## Methods

### Experimental setup

The laser source is a Ti:Sa laser system delivering linearly polarized ≈35 fs pulses at 800 nm with a Gaussian beam spatial profile, at a repetition rate of 10 Hz. An OV beam, carrying an orbital angular momentum (OAM) ℓ = ±1, is produced by a q-plate with a topological charge *q* = +1/2, as illustrated in Fig. 1. The upper left inset in Fig. 1(a) reports an image of the OV beam spatial intensity distribution as acquired by a beam profiler. It is characterised by an annular spatial profile, with a central region of zero intensity due to an undefined phase on the OV beam axis. The target is a single-crystalline Si (100) plate (dielectric constant ε_Si_ = 13.64 + 0.048*i* at 800 nm). The laser beam is focused by a lens (focal length 75 mm) onto the Si target sample mounted on a computer-controlled XY-translation stage at normal incidence. An electromechanical shutter controls the number of laser pulses, *N*, irradiated on the target surface. The morphological modifications of the irradiated surface are studied by using a field emission scanning electron microscope.

Figure 4(a) reports the fluence profile of the OV beam *Φ*(*r*) (normalized to its peak value *Φ*_*p*_) along a diameter of the annular beam (blue line), where *r* is the coordinate along the diameter. *Φ*(*r*) is well described by the following distribution (red line):


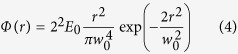


where *E*_*0*_ is the beam energy and *w*_*0*_ the waist of the corresponding fundamental Gaussian beam, derived from ref. 17. In our experiment *w*_*0*_ = (46 ± 2) μm. The peak fluence value is 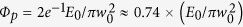 and is located at a radial distance 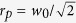 from the OV beam center at *r* = 0.

Figure 4(a) also illustrates the method used to determine the fluence threshold for the grooves formation *Φ*_*th,G*_: the values of the radii *r*_*G,in*_ and *r*_*G,ex*_ of the two circles delimiting the grooved area formed after irradiation with different values of the pulse number *N*, are measured on the SEM images of the irradiated region. Then, the respective values of the fluence are estimated from the OV beam spatial profile, and the fluence threshold *Φ*_*th,G*_ is evaluated as the average of the fluence values at *r*_*G,in*_ and *r*_*G,ex*_ with the corresponding uncertainty.

### Modeling

Our theoretical interpretation of the surface structure formation is based on a recent extension of the surface-scattered wave theory of Sipe *et al.*^21^ which takes into account the effects of the variation of the dielectric permittivity ε of the silicon target surface induced by laser pulse irradiation (for the details of this Sipe-Drude approach see Refs. 4, 22 and 23 e.g.).This approach allows to numerically calculate a spatial pattern of energy deposition on a rough target surface, and the formation of periodic surface structures is rationalized in terms of the spatial modulation induced by the interaction of the incoming radiation and the scattered surface wave. This is expressed by means of an “efficacy factor” *η*(***κ***) that is function of the characteristic wave-vector ***κ*** of the induced periodic structure in the Fourier spatial frequency domain (i.e. ***κ***  = 2*π*/Λ, Λ being the spatial period of the surface structure in the real space). For linearly-polarized laser pulses at normal incidence the main features of *η*(***κ***) are rather independent of the specific parameters used to describe the surface roughness^21^. Hence, it is typically depicted as spherically shaped islands and standard values of the Sipe theory for the shape (*s* = 0.4) and filling (*f* = 0.1) factors are exploited^5^,^6^,^23^. *η*(***κ***) is a two-dimensional (2D) map describing the modulation of laser energy distribution in the ***κ***-space, with wave vectors components *κ*_*x*_ and *κ*_*y*_ normal and parallel to the laser polarization, respectively. The map of *η*(***κ***) is symmetric with respect to *κ*_*x*_ = 0 and *κ*_*y*_ = 0. The variation of *ε* is modelled by exploiting two-temperature model and free-carrier number density equations^4^,^24^,^25^. In particular, during irradiation with a fixed pulse fluence *Φ*, the temporal variation of the free-carrier number density reaches a maximum value at a certain time t*. Consequently, at t* the real part of the dielectric permittivity reaches its minimum value. The value of the relative dielectric permittivity ε* at time t* is used in the simulations to calculate the efficacy factor distribution *η*(***κ***) according to Sipe theory. Finally, discrete 2D inverse Fourier transformation (2D-IFT) is exploited to convert the 2D map of the efficacy factor *η*(***κ***) into a corresponding map 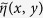 in the real (*x*,*y*) space (see e.g. Fig. 5), which allows a direct visualization of the energy modulation and an easier comparison with experimental observations.

## Additional Information

**How to cite this article**: JJ Nivas, J. *et al.* Direct Femtosecond Laser Surface Structuring with Optical Vortex Beams Generated by a q-plate. *Sci. Rep.* **5**, 17929; doi: 10.1038/srep17929 (2015).
